# New Appliances and Things Medical

**Published:** 1896-11-07

**Authors:** 


### NEW APPLIANCES AND THINGS MEDICAL.

[We shall be glad to receive, at our Office, 28 & 29, Southampton Street, Strand, London, W.C., from the manufacturers, specimens of all new preparations and appliances which may be brought out from time to time.]

### NEW DRUGS AND PREPARATIONS.

(Bleasdale, Limited, York.)

We have received a number of samples of drugs from the above firm. Some of them appear to us, after careful trial, to be of first-rate qualities; others, to which we have been unable to apply clinical tests, are undoubtedly, from a pharmaceutical point of view, most carefully prepared and of fine finish. We have here space to mention only a few of the samples sent us. One of the most interesting is a mixture which goes by the name of Neo-nitrite; it is intended as a substitute for the ordinary spirit. eth. nit. of the B.P. Though practically of the same composition as the latter, and possessing its therapeutic properties, it is very much cheaper as regards price, a matter of great importance to hospitals and institutions to whose notice we specially recommend it. Extract. Leonur. Cardiac. Liquid., a comparatively little-known preparation, is made from a rare British plant, said to possess extraordinary powers as a cardiac tonic, and one greatly valued by the old herbalists; it is also reported of great value in hepatic dropsy, chronic rheumatism, gout, and amenorrhœa. The dose is from half to two drachms. Liquor Cannabis Indic. is also a preparation worth noticing, as it appears to possess the beneficial effects of the Indian hemp, without the usual drawbacks in the way of cerebral excitement which is inseparably connected with the use of the ordinary tincture. Dr. Cowan Lees reports favourably of the aqueous preparation in contradistinction to the spiritous tincture. It may be used with advantage in certain cases of phthisis, indigestion, and constipation. Other preparations, which we have no space to mention in detail, but supplied by this firm, are: Boric. c. glyc., an antiseptic jelly for gynæcological use; Syr. violæ sicc., a dried syrup of violets, said to keep indefinitely, deeper in colour, and cheaper than the usual commercial varieties. Busy Mothers' Infants' Food; granular effervescent preparation, tasteless and coated pills.

